# Impact of comorbidity on patients with COVID-19 in India: A nationwide analysis

**DOI:** 10.3389/fpubh.2022.1027312

**Published:** 2023-01-27

**Authors:** Priya Singh, Yogendra Bhaskar, Pulkit Verma, Shweta Rana, Prabudh Goel, Sujeet Kumar, Krushna Chandra Gouda, Harpreet Singh

**Affiliations:** ^1^Division of Biomedical Informatics, Indian Council of Medical Research, New Delhi, India; ^2^Department of Paediatric Surgery, All India Institute of Medical Sciences (AIIMS), New Delhi, India; ^3^Centre for Proteomics and Drug Discovery, Amity University Maharashtra, Mumbai, India; ^4^Earth and Engineering Sciences Division, CSIR Fourth Paradigm Institute, Bangalore, India

**Keywords:** COVID-19, comorbidity, odd ratio, hypertension, epidemiology

## Abstract

**Background:**

The emergence of coronavirus disease (COVID-19) as a global pandemic has resulted in the loss of many lives and a significant decline in global economic losses. Thus, for a large country like India, there is a need to comprehend the dynamics of COVID-19 in a clustered way.

**Objective:**

To evaluate the clinical characteristics of patients with COVID-19 according to age, gender, and preexisting comorbidity. Patients with COVID-19 were categorized according to comorbidity, and the data over a 2-year period (1 January 2020 to 31 January 2022) were considered to analyze the impact of comorbidity on severe COVID-19 outcomes.

**Methods:**

For different age/gender groups, the distribution of COVID-19 positive, hospitalized, and mortality cases was estimated. The impact of comorbidity was assessed by computing incidence rate (IR), odds ratio (OR), and proportion analysis.

**Results:**

The results indicated that COVID-19 caused an exponential growth in mortality. In patients over the age of 50, the mortality rate was found to be very high, ~80%. Moreover, based on the estimation of OR, it can be inferred that age and various preexisting comorbidities were found to be predictors of severe COVID-19 outcomes. The strongest risk factors for COVID-19 mortality were preexisting comorbidities like diabetes (OR: 2.39; 95% confidence interval (CI): 2.31–2.47; *p* < 0.0001), hypertension (OR: 2.31; 95% CI: 2.23–2.39; *p* < 0.0001), and heart disease (OR: 2.19; 95% CI: 2.08–2.30; *p* < 0.0001). The proportion of fatal cases among patients positive for COVID-19 increased with the number of comorbidities.

**Conclusion:**

This study concluded that elderly patients with preexisting comorbidities were at an increased risk of COVID-19 mortality. Patients in the elderly age group with underlying medical conditions are recommended for preventive medical care or medical resources and vaccination against COVID-19.

## Introduction

Since its inception, coronavirus disease (COVID-19) has been in the limelight for a variety of reasons, including the fact that it caused human deaths and threatened the global healthcare system. Based on available data, ~380 million confirmed cases of COVID-19 and 5.8 million human deaths have been reported worldwide. Of these, India has witnessed 11% of all cases across the world and 8.7% of global mortality (1). Although several studies observed that patients infected with COVID-19 have underlying medical conditions, which are more likely to develop a severe illness and worse clinical outcomes (2–7), data on the association between COVID-19 and underlying comorbidities remain elusive. The patterns of clinical presentation and the ultimate outcomes have been found to be variable, and a correlation has been observed with the origin of the different variants of the coronavirus. However, those suffering from specific demographic variables and comorbidities have a higher incidence of hospitalization and mortality, consistent with all the variants known to date. Previous studies depicted a negative association between final outcomes with age (6, 8–11), gender (12–16), and comorbid conditions such as diabetes (17, 18), hypertension (19, 20), obesity (21, 22), heart disease (23–25), and chronic lung disease (26, 27). Moreover, global epidemiological studies showed that COVID-19 has gender differences in morbidity and mortality, with men showing higher morbidity and mortality than women (28–32). Different preexisting comorbidities affect the clinical course and final outcomes of a COVID-19 infection of different magnitudes (5, 6). Such information is, however, vital not only to formulate policies, addressing issues such as quarantine, leave-from-work, and treatment but also to enable the government to ensure optimal and appropriate resource allocation. Previous waves of the pandemic have brought the global healthcare infrastructure to its knees and have delivered a death blow to the demand-supply ratio.

Against this background, a nationwide analysis of the severity, clinical course, and outcomes of a COVID-19 infection in patients with preexisting comorbidities and specific age-group impressions in India is presented in this study.

## Data and methods

### Study design

**Figure 1:**
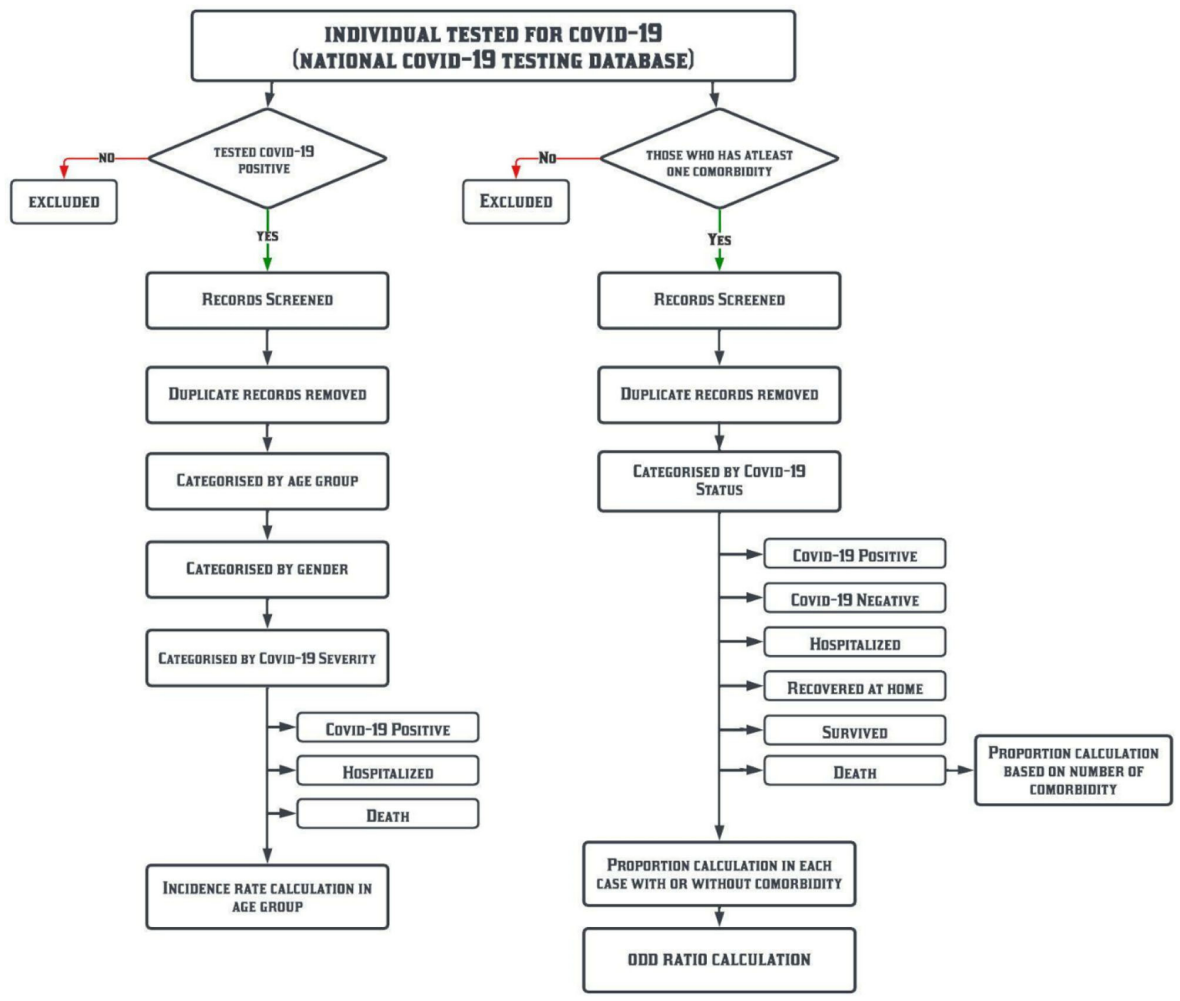
Data analysis work flow.

This is a pan-India retrospective study based on the COVID-19 management data from the first positive case reported till 31 January 2022. The data analysis workflow is shown in Figure 1.

### Data source

(a) The data repository of the Indian Council of Medical Research (ICMR) was developed during the pandemic management in the study timeframe and (b) the specimen referral forms (SRFs), which were filled for demographic and clinical data (name, age, gender, address, occupation, comorbidities, symptoms, date of sample tested, confirmation date, date of hospitalization, history of travel, etc.), were mandatory at the time of testing for COVID-19 across all laboratories, hospitals, booths, and campuses in the country (https://www.icmr.gov.in/pdf/covid/labs/Revised_SRF_Form_06012022.pdf) (Supplementary material S1).

Each patient was subsequently assigned a SRF ID, which might be used for patient identification in all COVID-19 care centres in India.

Comorbidities included in this study are mainly diabetes, hypertension, obesity, heart disease, and chronic lung disease.

### Statistical analysis

We estimated the prevalence of all COVID-19 positive cases and severe outcomes among them, classified by age and gender. For each age group, chi-squared statistics were applied to test the impact of gender on the severity of COVID-19 (hospitalizations and deaths). For the purpose of this study, the need for hospitalization and death of patients infected with COVID-19 have been represented as two severe outcomes. The incidence rate (IR) was calculated based on the population of India in the year 2011, i.e., 1,210,854,977 (121 crores) per 100,000 people for COVID-19 positive, hospitalized, and death cases. Subsequently, a subgroup analysis was performed based on the incidence of the disease in specific age groups of the population. Age- and gender-specific information was derived from the census data of the year 2011 (33). The proportion of patients with comorbidities (any one of the five listed above) was calculated among COVID-19 positive cases who were managed at home, those who required hospitalization, and those who succumbed to the disease.

The odds ratio (OR) technique was performed using the statistical software SPSS. In patients with and without preexisting comorbidities, the odds of COVID-19 positivity and its outcomes were calculated. The 95% confidence interval (CI) and *p*-value for each OR were tabulated, with a significance level of *p* of < 0.05.

The OR (Equation 1) was calculated using the following equation:


(1) $$\begin{array}{l} Odds\;ratio=\frac{A^{*}D}{B^{*}C}, \end{array}$$

where *A* is the number of patients infected with COVID-19 and having a preexisting comorbidity, *B* is the number of patients having a preexisting comorbidity and no COVID-19 infection, *C* is the number of patients infected with COVID-19 and having no preexisting comorbidities, and *D* is the number of patients having no COVID-19 infection and preexisting comorbidities.

An OR of <1 indicates that preexisting comorbidities are not a major factor affecting patients infected with COVID-19. Higher OR values simultaneously increase the risk factors for patient mortality. An OR value of >1 indicates that preexisting comorbidities may be a major factor affecting patients infected with COVID-19 (34, 35). By considering specific age groups and the number of preexisting comorbidities, the proportion of fatal cases of patients infected with COVID-19 has also been analyzed.

## Results

### Characteristics of patients with COVID-19 according to age

In India, a total of 40,691,059 COVID-19 cases were observed over a 25-month period (1 January 2020 to 31 January 2022), of which 3,442,018 (8.5%) were hospitalized and 286,962 (0.7%) died (Table 1). The data were divided into specific age groups (Table 1), and the following inferences were drawn from the analysis: (i) a total of 57,922 infants (0–11 months) were found to be COVID-19 positive and only 0.06% were reported to have died; (ii) in the under-20 age group, 4,584,714 COVID-19 positive cases were reported, and, a low morality rate of 0.53% was reported similar to infants; (iii) in the 20–49 age group, the COVID-19 positivity (60%) and hospitalization (58%) were high but the fatality (20%) was low, and (iv) the mortality rate (~80%) was very high among those over the age of 50 despite very low hospitalization (32%) and positivity (29%). The data showed that older age may increase the risk of hospitalization and death in some cases, i.e., an increase in trends between age and severe outcomes among patients positive for COVID-19.

**Table 1:** Characteristics of patients with COVID-19 in diverse age groups.

**Age category (years)**	**COVID-19 positive** ***n* = 40,691,059 (100%)**	**Hospitalized** ***n* = 3,442,018 (100%)**	**Death** ***n* = 286,962 (100%)**
Infant	57,922 (0.14%)	4,857 (0.14%)	174 (0.06%)
1–9	1,230,711 (3.02%)	97,807 (2.84%)	425 (0.15%)
10–19	3,354,004 (8.24%)	248,712 (7.23%)	1,100 (0.38%)
20–29	8,289,655 (20.37%)	629,408 (18.29%)	5,752 (2%)
30–39	8,968,608 (22.04%)	752,124 (21.85%)	17,163 (5.98%)
40–49	6,972,306 (17.13%)	613,518 (17.82%)	35,341 (12.32%)
50–59	5,652,153 (13.89%)	509,040 (14.79%)	60,275 (21%)
60–69	3,799,581 (9.34%)	362,531 (10.53%)	79,071 (27.55%)
70–79	1,764,213 (4.34%)	171,350 (4.98%)	59,415 (20.70%)
80–89	521,026 (1.28%)	45,628 (1.33%)	24,204 (8.43%)
90+	80,880 (0.20%)	7,043 (0.20%)	4,042 (1.41%)
Total	40,691,059	3,442,018	286,962

**Figure 2:**
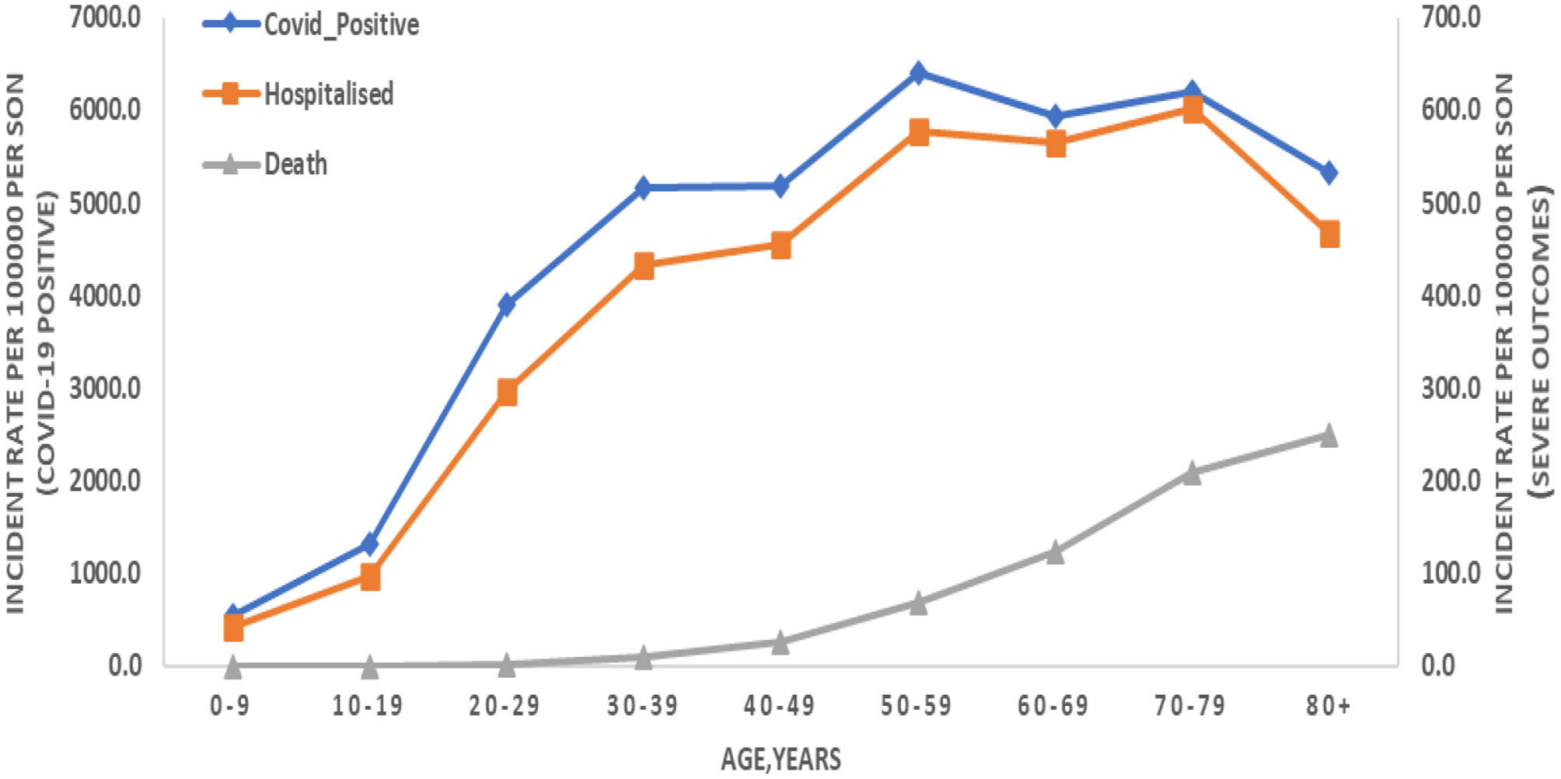
Incidence rate (IR) per 100,000 people infected with COVID-19 and their severe outcomes (hospitalized and death) in the specific age group. COVID-19 cases are presented on the left-hand side of *Y*-axis and severe outcomes on the right-hand side of *Y*-axis.

To better understand the scope of the risk of infections, IR was calculated per 10^5^ of the total population. IR for all COVID-19 positive, hospitalized, and death cases was found to be 3,360.5, 284.3, and 23.7, respectively. IR has also been estimated in specific age groups, and the following key points can be inferred (Figure 2): (i) the IR for COVID-19 positive cases in infants (0–11 months) was 537.5, while the death IR was very low, i.e., 0.24, (ii), the IR for COVID-19 positive cases was 1,324.5 in those below 20 years of age and a very low death IR of 0.43 was observed similar to infants, (iii) moreover, IR showed an increasing trend with the increasing age group for COVID-19 hospitalization, while an exponential growth in death IR was observed. Thus, IR data showed the risk of infections (and deaths) among patients with COVID-19 positive increases with age. The risk of deaths and rapid infection has been found to be more pronounced among older people in India, regardless of rural or urban.

### Characteristics of patients with COVID-19 according to gender

The proportion of risk among patients also varies widely when analyzed on the basis of gender. COVID-19 infections and their severe outcomes were more common among men in the 30–39 age group compared with women (Table 2). In the 30–39 age group, male patients were more affected than female patients in COVID-19 positive cases (62% men; 38% women), and similar results were obtained in hospitalized (64% men; 36% women) and death (72% men; 28% women) cases, as presented in Table 2. Moreover, while taking an average, a similar pattern can be seen in the 20–69 age group, i.e., male patients are more severely affected by the COVID-19 outcome than female patients (Table 2). These data also showed that men were infected than women, which could be due to the fact that, in the 20–69 age group, the working population of India comprised more men than women. To validate the impact of gender on the severity of COVID-19 (hospitalizations and deaths), a chi-squared test was used. The results showed that, for most age groups, men and women had a statistically significant relationship (*p* < 0.05) with the severity of COVID-19 (Table 2).

**Table 2:** Characteristics of patients with COVID-19 according to gender.

**Age category**	**COVID-19 positive**		**Hospitalized**			**Death**		

	**Male subjects**	**Female subjects**	**Male subjects**	**Female subjects**	**Chi-square (** ***P*** **-value)**	**Male subjects**	**Female subjects**	**Chi-square (** ***P*** **-Value)**
Infant	32,422 (56%)	25,500 (44%)	2,795 (58%)	2,062 (42%)	5.23 (0.022)	97 (56%)	77 (44%)	0.01 (0.987)
0-9	663,464 (54%)	566,640 (46%)	53,118 (54%)	44,665 (46%)	6.37 (0.011)	272 (64%)	153 (36%)	16.93 (0.001)
10-19	1,859,655 (55%)	1,493,623 (45%)	141,900 (57%)	106,762 (43%)	280.91 (0.001)	595 (54%)	505 (46%)	0.77 (0.377)
20-29	4,863,748 (59%)	3,424,344 (41%)	381,877 (61%)	247,425 (39%)	1,122.36 (0.001)	3,562 (62%)	2,189 (38%)	24.99 (0.001)
30-39	5,574,392 (62%)	3,392,564 (38%)	484,538 (64%)	267,434 (36%)	1,797.7 (0.001)	12,324 (72%)	4,833 (28%)	682.27 (0.001)
40-49	4,220,769 (61%)	2,750,252 (39%)	387,151 (63%)	226,250 (37%)	1,856.86 (0.001)	24,083 (68%)	11,251 (32%)	860.84 (0.001)
50-59	3,323,505 (59%)	2,327,632 (41%)	311,917 (61%)	197,033 (39%)	1,414.42 (0.001)	39,339 (65%)	20,921 (35%)	1,052.58 (0.001)
60-69	2,191,686 (58%)	1,607,215 (42%)	211,645 (58%)	150,805 (42%)	80.46 (0.001)	50,661 (64%)	28,396 (36%)	1,349.93 (0.001)
70-79	1,049,700 (59%)	714,205 (40%)	103,857 (61%)	67,464 (39%)	97.2 (0.001)	39,533 (67%)	19,875 (33%)	1,262.4 (0.001)
80-89	302,475 (58%)	218,457 (42%)	27,561 (60%)	18,059 (40%)	113.29 (0.001)	16,409 (68%)	7,792 (32%)	988.15 (0.001)
90+	41,778 (52%)	39,051 (48%)	3,638 (52%)	3,405 (48%)	0.002 (0.964)	2,509 (62%)	1,533 (38%)	183.36 (0.001)
Total	41,778 (59%)	16,559,483 (41%)	2,109,997 (61%)	1,331,364 (39%)	6,333.36 (0.001)	189,384 (66%)	97,525 (34%)	5,393.09 (0.001)

The impact of gender on the severity of COVID-19 is presented in terms of chi-squared statistics and p-value.

### Presence of comorbidities and the clinical characteristics and outcomes of COVID-19

**Figure 3:**
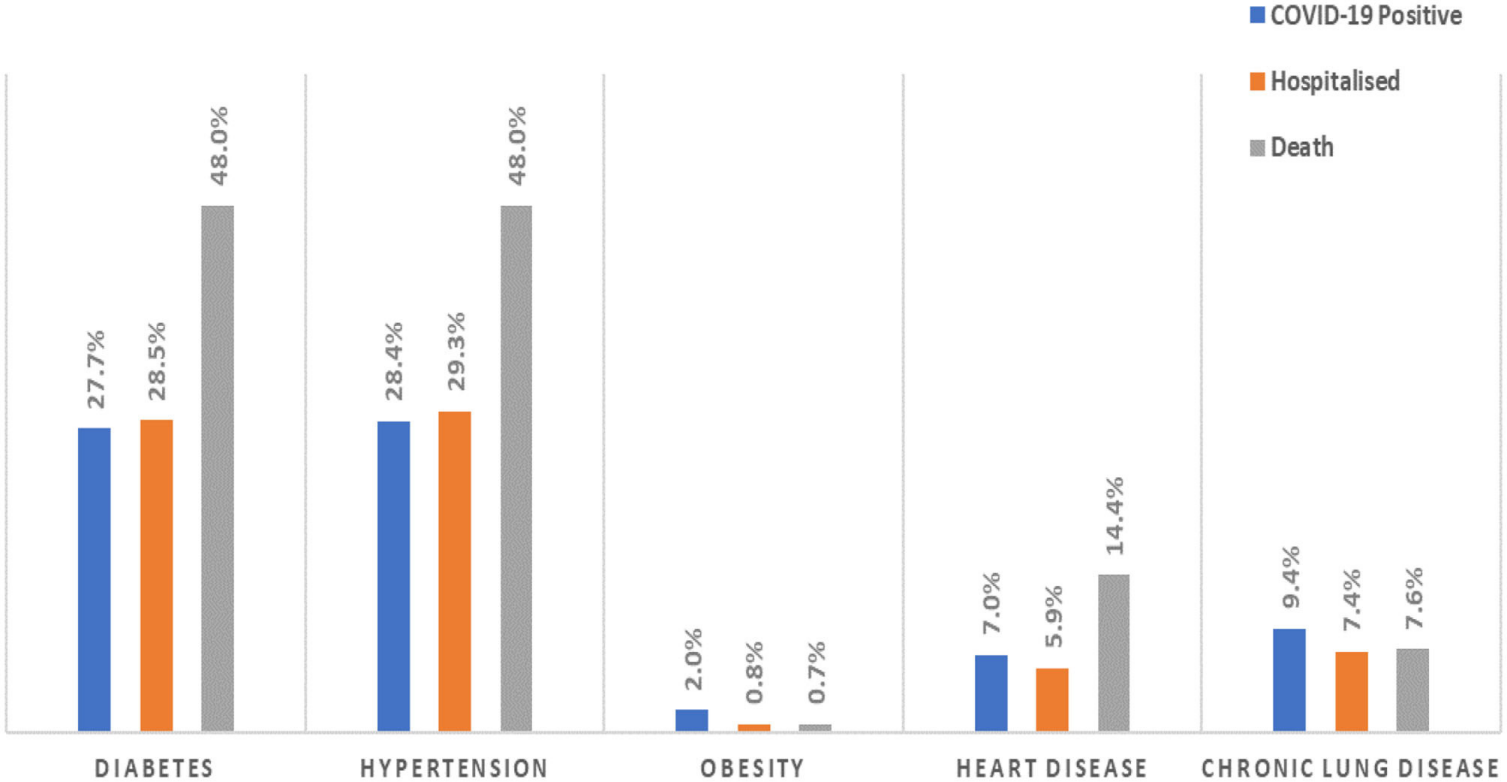
The proportion of patients with comorbidities among patients positive for COVID-19 and their severe outcomes (hospitalized and death).

We analyzed how five major comorbidities, namely, diabetes, hypertension, obesity, heart disease, and chronic lung disease, affected a COVID-19 infection and its severe outcomes in individuals. Of the 661,894,210 cases tested, 5,817,884 patients had at least one comorbidity, of which 563,233 were COVID-19 positive. Considering all positive cases (563,233), we observed that 62,185 (11%) and 13,877 (2.5%) were hospitalized and lost their lives, respectively. Moreover, the distribution of COVID-19 positive, hospitalized, and mortality cases in the five comorbidities was as follows: (i) the major risk factors for COVID-19 death were similar to the major risk factors for diabetes and hypertension (6,661 cases, 48%), (ii) an increased risk of mortality was also associated with heart disease (1,993 cases, 14.4%), chronic lung disease (1,055 cases, 7.6%), and obesity (103 cases, 0.7%), as shown in Figure 3, (iii) the risk factors for COVID-19 hospitalization were hypertension (18,235 cases, 29.3%) and diabetes (17,698 cases, 28.5%), (iv) chronic lung disease (4,627 cases, 7.4%), heart disease (3,667 cases, 5.9%), and obesity (491 cases, 0.8%), which also increased the risk of hospitalization (Figure 3), (v) the major risk factors for COVID-19 cases were hypertension (160,114 cases, 28.4%) and diabetes (156,155 cases, 27.7%), (vi) chronic lung disease (53,092 cases, 9.4%), heart disease (39,516 cases, 7%), and obesity (11,521 cases, 2%), which also increased the risk of COVID-19 (Figure 3). The data indicated increased severity of COVID-19 in patients with at least one preexisting comorbidity.

### Predictors of severe COVID-19 outcomes

**Figure 4:**
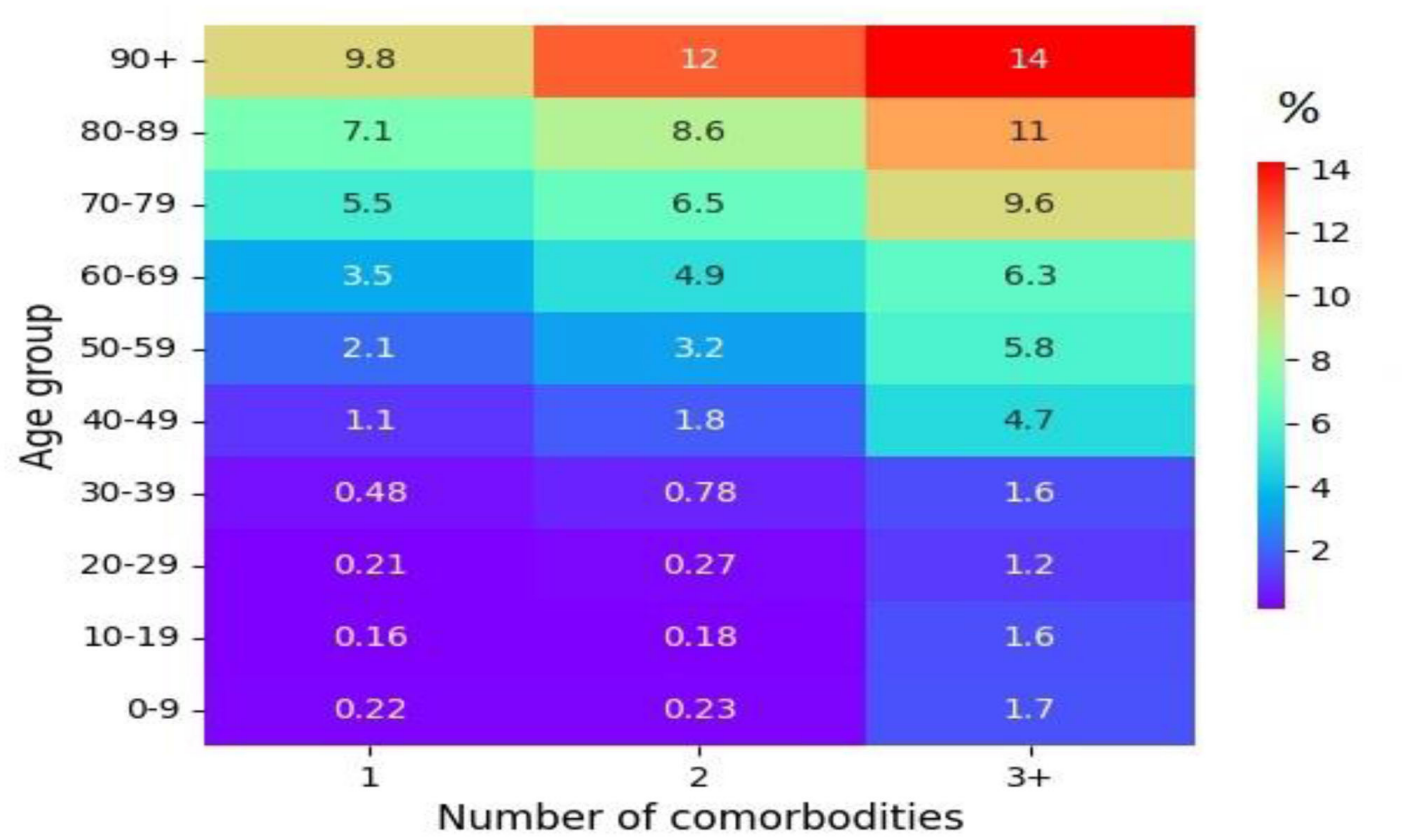
The proportion (%) of fatal outcomes in cases of a COVID-19 infection by age groups and the number of comorbidities.

To analyze the impact of the five major comorbidities on cases infected with COVID-19 and their severe outcomes, statistical analyses, such as OR, were computed, which can provide better guidance in predicting COVID-19 outcomes. We observed that many comorbidities were risk factors for mortality. The risk of a severe COVID-19 outcome increased in tandem with the number of comorbidities. The more comorbidities a person has, the greater the risk of a severe COVID-19 infection. In this study, we analyzed the OR of the five most common comorbidities to compare COVID-19 cases and their severe outcomes in people with and without preexisting comorbidities. The strongest risk factors for COVID-19 death were observed in patients with diabetes (OR: 2.39; 95% CI: 2.31–2.47; *p* < 0.0001), hypertension (OR: 2.31; 95% CI: 2.23–2.39; *p* < 0.0001), and heart disease (OR: 2.19; 95% CI: 2.08–2.30; *p* < 0.0001) when comparing the odds between people with and without preexisting comorbidities, as presented in Table 3. However, chronic lung disease (OR: 0.80; 95% CI: 0.74–0.85; *p* < 0.0001) and obesity (OR: 0.35; 95% CI: 0.29–0.43; *p* < 0.0001) were also associated with the risk of COVID-19 death. The risk factors for COVID-19 hospitalization were hypertension (OR: 1.02; 95% CI: 0.99–1.0; *p* = 0.09923), diabetes 1.01 (95% CI: 0.99–1.03; *p* = 0.5222), heart disease (OR: 0.78; 95% CI: 0.75–0.80; *p* < 0.0001), and chronic lung disease (OR: 0.76; 95% CI: 0.73–0.78; *p* < 0.0001) (Table 3). Moreover, similar to mortality risk, obesity (OR: 0.36; 95% CI: 0.32–0.39; *p* < 0.0001) is also not a major factor in the risk of hospitalization. The major risks for COVID-19 positivity were diabetes (OR: 2.67; 95% CI: 2.65–2.69; *p* < 0.0001) and hypertension (OR: 2.40; 95% CI: 2.39–2.42; *p* < 0.0001), as presented in Table 3. Similarly, heart disease (OR: 1.41; 95% CI: 1.39–1.42; *p* < 0.0001), obesity (OR: 1.23; 95% CI: 1.20–1.25; *p* < 0.0001), and chronic lung disease (OR: 0.98; 95% CI: 0.97–0.99; *p* = 0.0001) also increased the risk of COVID-19 positivity. Moreover, the proportion analysis of COVID-19 cases showed that older patients with a larger number of comorbidities were more likely to have severe outcomes and mortality (Figure 4). With three or more comorbidities, the proportion of fatal cases varied from 4.7% (40–49 years) to 14% (over the age 90 years). For the age group above 50 years, 28% of fatal cases had at least one, 36% of fatal cases had at least two, and 47% of fatal cases had three or more preexisting comorbidities. For all age groups, the more comorbidities designate a higher risk of mortality among the infected. Therefore, the risk of severe COVID-19 outcomes in a patient infected with COVID-19 increased with age, and severe COVID-19 outcomes were associated with the severity of preexisting comorbidities.

**Table 3:** Relative risks of COVID-19 positivity and its severe outcomes with individual comorbidity are presented in terms of odds ratios (ORs) with 95% confidence interval (CI 95%) and *p*-value.

**Comorbidities**	**COVID-19 positive**	**Hospitalized**	**Death**
Diabetes	2.67 (95% CI: 2.65–2.69; *p < * 0.0001)	1.01 (95% CI: 0.99–1.03; *P = p =* 0.5222)	2.39 (95% CI: 2.31–2.47; *P < * 0.0001)
Hypertension	2.40 (95% CI: 2.39–2.42; *P < * 0.0001)	1.02 (95% CI: 0.99–1.0; *P =* 0.09923)	2.31 (95% CI: 2.23–2.39; *P < * 0.0001)
Obesity	1.23 (95% CI: 1.20–1.25; *P < * 0.0001)	0.36 (95% CI: 0.32–0.39; *P < * 0.0001)	0.35 (95% CI: 0.29–0.43; *P < * 0.0001)
Heart disease	1.41 (95% CI: 1.39–1.42; *P < * 0.0001)	0.78 (95% CI: 0.75–0.80; *P < * 0.0001)	2.19 (95% CI: 2.08–2.30; *P < * 0.0001)
Chronic lung disease	0.98 (95% CI: 0.97–0.99; *P =* 0.0001)	0.76 (95% CI: 0.73–0.78; *P < * 0.0001)	0.80 (95% CI: 0.74–0.85; *P < * 0.0001)

This study systematically evaluated the effects of comorbidities on the clinical features and prognosis of patients with COVID-19 on a national scale. Patients with COVID-19 seemed to have a higher rate of endocrine comorbidities. Patients with at least, if not more than, one comorbidity showed worse clinical outcomes with a serious infection. With a large sample size and extensive coverage of geographic locations across India, these findings provided more factual evidence that baseline underlying medical conditions should be considered in a comprehensive risk analysis of the prognosis of patients hospitalized with COVID-19.

## Discussion and conclusions

In this study, of the five comorbidities, hypertension has been identified as the most common comorbidity, resulting in a severe COVID-19 infection and hospitalization, followed by diabetes, chronic lung disease, heart disease, and obesity. However, from the perspective of an increased risk of death, diabetes has been identified as the most common comorbidity, followed by hypertension, heart disease, chronic lung disease, and obesity. Moreover, a meta-analysis of the relative risk of COVID-19 positivity in terms of ORs depicts diabetes (2.67, 95% CI: 2.65–2.68) as the highest relative risk, followed by hypertension (2.40, 95% CI: 2.39–2.42), obesity (1.23, 95% CI: 1.20–1.25), heart disease (1.41, 95% CI: 1.40–1.43), and chronic lung disease (0.98, 95% CI: 0.97–0.99). Meanwhile, in terms of the COVID-19 severe outcomes, i.e., hospitalization and death, the relative risk in terms of ORs depicts diabetes as the highest relative risk, followed by hypertension, heart disease, chronic lung disease, and obesity. The abovementioned outcomes of a higher relative risk associated with diabetes and hypertension may correlate with a recent study regarding COVID-19-related thrombotic complications, showing an incidence of ~49% (36). The same has been reinforced and highlighted in a retrospective population-based cohort study conducted by the Taiwan Ministry of Health and Welfare Clinical Trial Centre and also in a separate study funded by the Beijing Municipal Science and Technology Commission of China. These studies confirm a direct relationship between venous thromboembolism (VTE) and hypertension and diabetes. VTE cases were substantially higher in patients with hypertension or diabetes compared with patients without hypertension or diabetes (37, 38). Our findings therefore suggest a spectrum of comorbidities in patients with COVID-19 in the existing literature based on the larger sample sizes and representativeness of the overall patient population in India.

A gender-based bias between the number of infections, hospitalizations, and deaths is clearly observed among men and women. For example, a higher prevalence of positivity, hospitalizations, and deaths has been observed among the working-age male population compared to the female population (39). This gender-based bias was evident in the outcome when correlated with the findings on Periodic Labor Force Participation (PLFS) of 2020–2021 for urban, the Labor Force Participation Rate (LFPR), and the Worker Population Ratio (WPR), which indicated a stark difference between the percentage of the male population who were available and actually working in the same age range compared to the female population. The average LFPR for the years 2020–2021 among men and women is 73.2 and 20.7%, respectively, while the average WPR for the same period among men and women is 64.48 and 17.76%, respectively [PLFS]. The abovementioned data provide a suitable reason for the possibility of a gender-based bias among COVID-19 infected cases, as the percentage of the working female population is relatively low when compared to men.

Furthermore, according to our analysis, the highest number of positive cases seemed to occur in the 20–59 age group, with the highest positivity and severity in the 30–39 age group. As the 15–59 age group constitutes the working-age population of any country, and based on the SRS Statistical Report 2018 (the annual publication of the office of the census commissioner and registrar general), two-thirds of the population of India are in the 15–59 age group. This could explain the high IR of COVID-19 positivity in the 20–59 age group, which is above 3,000.

In terms of the severity of the disease in elderly patients, our analysis depicts a high death rate of ~80%, in the population over the age of 50. This is also clearly evident from the IR (Figure 2), which shows an increase in the incidence of positivity, hospitalization, and death in the 50–80 age group. Sustained higher severity within the abovementioned age range (i.e., 50–80) could be due to the individuals being in the working-age population, a higher number of comorbidities, and immune system-related changes caused by an overreactive but ineffective immune alert system (40), all of which translated to the above-average severity of COVID-19 outcomes among the elderly.

A few limitations of this study deserve attention. Although not the first of its type, the study cohort is largely available in the literature (*n* = 40,691,059 COVID-19 cases observed over a 25-month period). Data availability was limited to information provided by the subject in a designated form when seeking a COVID-19 test. There might be patients who succumbed to diseases at home and never went to any health facility. Last but not least, the availability of follow-up data in this study is limited. Despite these limitations, this study would be helpful when designing specific guidelines and standard operating procedures for people with underlying comorbidities to mitigate the number of fatalities. Moreover, early classifications based on a prospective study of severe and non-severe cases of COVID-19 depending on the underlying number of comorbidities will result in improved patient outcomes as well as decreased hospitalization and deaths.

## Data availability statement

The raw data supporting the conclusions of this article will be made available by the authors, without undue reservation.

## Author contributions

PS, KG, and HS designed, implemented, and wrote the manuscript. YB and PV wrote and reviewed the manuscript. SR edited the manuscript. PG, SK, and KG reviewed and edited the manuscript. HS coordinated the study, provided resources and facilities, reviewed, and edited the manuscript. All listed authors contributed substantially, directly, and intellectually to the work and approved it for publication.
