# Supplementary material for: Impact of comorbidity on patients with COVID-19 in India: A nationwide analysis
Source: Front Public Health. 2023 Jan 27;10:1027312. doi: 10.3389/fpubh.2022.1027312 (PMC9911546; doi:10.3389/fpubh.2022.1027312)
Supplement: Supplementary file 1 [file Data_Sheet_1.PDF]

ICMR Specimen Referral Form for COVID-19 (SARS-CoV2)

INTRODUCTION

This form is for collection centres/ labs to enter details of the samples being tested for Covid-19. It is mandatory to fill this form for each and every sample being tested. It is essential that the collection centres/ labs exercise caution to ensure that correct information is captured in the form.

INSTRUCTIONS

- Inform the local / district / state health authorities, especially surveillance officer for further guidance
- Seek guidance on requirements for the clinical specimen collection and transport from nodal officer
- This form may be filled in and shared with the IDSP and forwarded to a lab where testing is planned
- Fields marked with asterisk (*) are mandatory

SECTION A – PATIENT DETAILS

A.1 TEST INITIATION DETAILS

***Sample collected first time:** **Yes** ☐ **No** ☐

If No, Patient ID: …………………………………………

A.2 PERSONAL DETAILS

***Patient Name:** ………………………………………………. **Father's Name**…………………………………………………….

***Age: ……. Years/Months/ Days (If age <1 yr, pls. tick months/ days checkbox)**

*** Gender: Male** ☐ **Female** ☐ **Transgender** ☐

***Occupation: Health Care Worker** ☐ **Police** ☐ **Sanitation** ☐ **Security Guards** ☐ **Others** ☐

***Mobile Number:** ☐☐☐☐☐☐☐☐☐☐☐ **Mobile Number belongs to: Patient** ☐ **Family** ☐

***Nationality:** ……………………………………….

***Present patient address:** ……………………………. ***Downloaded Aarogya Setu App: Yes** ☐ **No** ☐

Pincode: ……………………………………………… ***Location: Urban** ☐ **Rural** ☐ **Tribal** ☐ **(Select either of the ones)**

***District ………………………………………………..** ***State** :…………………………………………………………………………..

(These fields to be filled for all patients including foreigners)

Aadhar No. (For Indians): ……………………………………………………………….

Passport No. (For Foreign Nationals): ……………………..

***Received COVID-19 vaccine Yes** ☐ **No** ☐

***CoWIN Beneficiary ID (If Available, else leave it Blank)…………………………………………………………………**

***If yes type of vaccine (in drop down)**

		**Covaxin (Bharat Biotech)**	☐	**Covishield (Serum Institute of India)**	☐
**Sputnik V (The Gamaleya National Center)**	☐	**BNT162b2 (Pfizer-BioNTech)**	☐	**mRNA-1273 (Moderna)**	☐
**Ad26.COV2.5 (Johnson & Johnson)**	☐	**Covovax (Serum Institute of India)**	☐	**ZyCoV-D (Zydus Cadila)**	☐
**NVX-CoV2373 (Novavax)**	☐	**Covilo (SinoPharm)**	☐	**CoronaVac (Sinovac)**	☐

***Date of Dose 1 -----/-----/----------** ***Dose 2 received? – Yes/ No (Mandatory)** **If yes, Date of Dose 2 -----/-----/--------- (Mandatory)**

*A.3 SPECIMEN INFORMATION FROM REFERRING AGENCY

***Specimen type:** Throat Swab ☐ Nasal Swab ☐ Bronchoalveolar lavage ☐ Endotracheal Aspirate ☐ Nasopharyngeal swab ☐

***Type of test RT-PCR ☐ Rapid Antigen Test (RAT) ☐**

***Name of kit used: …………………..................................**

***Collection date: --/--/----**

***Sample ID (Label) ………………………………………….**

Symptomatic ☐ Asymptomatic ☐

Contact of a lab confirmed case: Yes ☐ No ☐

If, RT-PCR test, name of lab where sample is sent for testing (Drop down – list of RT-PCR/ TrueNat/ CBNAAT labs)

*** Mode of Transport used to visit testing facility**

☐ **Public – In drop down menu – Bus, Metro, Train, Cab, Auto, Ambulance**

☐ **Private – In drop down menu – Car, Scooty, Bike, Bicycle, Walk**

☐ **Not Applicable**

Please Note - Hospital form is required for the patients visiting OPD, IPD and Emergency and Community form is required for patients under containment zone/ Non-containment area/ Point of entry/ Testing on demand

*A.3.1 For Community

Sample collected from (In Dropdown) - **Containment Zone/Non-containment area/Point of entry (Select either of the ones)**

Cat 1: All symptomatic (ILI symptoms) cases

Cat 2: All asymptomatic high-risk individuals (Any individual who falls under Section B2)
Cat 3: All symptomatic (ILI symptoms) individuals with history of international travel in the last 14 days
Cat 4: All individuals who wish to get themselves tested

A.3.2 For Hospital

Cat 1: All patients of Severe Acute Respiratory Infection (SARI)
Cat 2: All symptomatic (ILI symptoms) patients presenting in a healthcare setting
Cat 3: Asymptomatic high-risk patients who are hospitalized or seeking immediate hospitalization
Cat 4: Asymptomatic patients undergoing surgical / non-surgical invasive procedures (not to be tested more than once a week during hospital stay).
Cat 5: All pregnant women in/near labour who are hospitalized for delivery
Cat 6: All symptomatic neonates presenting with acute respiratory / sepsis like illness
Cat 7: Patients presenting with atypical manifestations [stroke, encephalitis, pulmonary embolism, acute coronary symptoms, Guillain Barre syndrome, Multi-system Inflammatory Syndrome in Children (MIS-C), progressive gastrointestinal symptoms] based on the discretion of the treating physician
Cat 8: All individuals who wish to get themselves tested

**Fields marked with asterisk are mandatory to be filled*

Please Note: Section B1 and B2 need to be filled for both Community and Hospital settings. Section B3 needs to be filled only for Hospital settings

SECTION B- MEDICAL INFORMATION

B.1 CLINICAL SYMPTOMS AND SIGNS

Cough ☐ **Loss of taste** ☐

Sore Throat ☐ **Diarrhoea** ☐

Fever ☐ **Breathlessness** ☐

Loss of smell **Other symptoms, please specify:** ____________________

ate of onset of First Symptom(dd/mm/yy): ☐☐ ☐☐ ☐☐

B.2 PRE-EXISTING MEDICAL CONDITIONS

Diabetes ☐ **Over weight/ Obesity** ☐

Heart disease ☐ **Hypertension** ☐

Chronic Lung disease ☐ **Cancer** ☐

Chronic Kidney Disease ☐ **Any other please specify:** ____________________

B.3 HOSPITALIZATION DETAILS

Hospitalized: Yes ☐ **No** ☐

Hospital State: ………………………………………………… **Hospital District:** ……………………………………………….

Hospitalization Date: ☐☐ ☐☐ ☐☐

Hospital Name: …………………………………………………

TEST RESULT (To be filled by Covid-19 testing lab facility)

Date of sample receipt(dd/mm/yy)	Sample accepted/ Rejected	Date of Testing (dd/mm/yy)	Test result (Positive / Negative)	Repeat Sample required (Yes / No)	Sign of Authority (Lab in charge)
